# Increased plasma levels of IL-6 are associated with striatal structural atrophy in major depressive disorder patients with anhedonia

**DOI:** 10.3389/fpsyt.2022.1016735

**Published:** 2022-11-03

**Authors:** Shaojia Lu, Congchong Wu, Lili Jia, Zhe Fang, Jing Lu, Tingting Mou, Shaohua Hu, Hongjian He, Manli Huang, Yi Xu

**Affiliations:** ^1^Department of Psychiatry, The First Affiliated Hospital, Zhejiang University School of Medicine, Key Laboratory of Mental Disorder's Management of Zhejiang Province, Zhejiang Engineering Center for Mathematical Mental Health, Hangzhou, China; ^2^Faculty of Clinical Medicine, Zhejiang University School of Medicine, Hangzhou, China; ^3^Department of Clinical Psychology, The Fifth Peoples' Hospital of Lin'an District, Hangzhou, China; ^4^College of Biomedical Engineering and Instrument Science, Center for Brain Imaging Science and Technology, Zhejiang University, Hangzhou, China

**Keywords:** major depressive disorder, anhedonia, interleukin-6, gray matter volume, striatum

## Abstract

**Background:**

Anhedonia, as the core endophenotype of major depressive disorder (MDD), is closely related to poor prognosis, but the mechanism of this feature remains to be understood. The aim of this study was to investigate the inflammatory factors and brain structural alterations in MDD patients with anhedonia and evaluate the relationship between these factors.

**Methods:**

We assessed the plasma levels of interleukin-1 beta (IL-1β), interleukin-6 (IL-6) and tumor necrosis factor alpha (TNF-α) in MDD patients with anhedonia (*n* = 22), MDD patients without anhedonia (*n* = 20), and age- and sex-matched healthy controls (HCs, *n* = 20) by enzyme-linked immunosorbent assay kits. All participants underwent high-resolution brain magnetic resonance imaging (MRI) scans, and voxel-based morphometry (VBM) was used to evaluate their gray matter volume (GMV). We compared inflammatory factors and GMV among the three groups and explored their relationships in MDD patients with anhedonia.

**Results:**

Compared with those of HCs, plasma levels of IL-1β were increased in patients with MDD independent of anhedonia features, while plasma levels of IL-6 were elevated in MDD patients with anhedonia only. Meanwhile, MDD patients with anhedonia exhibited reduced GMV in the left striatal structures compared to MDD patients without anhedonia and HCs. Moreover, a significant association was observed between increased plasma levels of IL-6 and decreased GMV of the left putamen in MDD patients with anhedonia.

**Conclusions:**

The present research outcomes suggest that anhedonia is associated with increased plasma levels of IL-6 and decreased GMV in the left striatal structures. In addition, this study demonstrates that GMV loss in the left putamen is related to increased plasma levels of IL-6 in MDD with anhedonia, which provides further insights into the possible mechanisms of anhedonia.

## Introduction

Major depressive disorder (MDD) is a highly prevalent and debilitating mental condition that causes a significant disease burden to families and world society (1). According to a recent national epidemiological survey of mental disorders conducted in China, the lifetime prevalence of MDD is approximately 3.4% (2). To date, clinical depression is still critical for the diagnosis of MDD (3) as the potential pathogenesis and etiology underlying depression remain unclear (4). In the past few decades, numerous studies have been developed that reveal the pathophysiology of depression. MDD patients, specifically, have been demonstrated to exhibit disturbances of the inflammatory system (5) and volumetric alterations in certain brain structures (6). However, the current findings are inconsistent and controversial because of the different clinical manifestations implicated in MDD (7). In this context, studies to identify clusters of depressive symptoms and corresponding biomarkers have been conducted, which may be beneficial for characterizing depression phenotypes and individualized diagnosis and treatment.

Anhedonia, defined as a loss of interest and/or the capacity to experience pleasure in previously enjoyed activities, is a core feature of MDD. It has been revealed that anhedonia is a strong predictor for poor response to antidepressant treatment (8), increased suicidal behavior (9), and distinct impairments of overall functioning (10) in depressed patients. Recent works have revealed that MDD with anhedonia may be treated as a functional subtype of depression with a unique clinical prognosis and biological alterations (11, 12). Furthermore, convergent lines of research also suggest that anhedonia acts as an endophenotypic marker and as a risk factor for MDD (13, 14). Therefore, clarifying the neural mechanism of anhedonia is both necessary and important in understanding the pathophysiology of MDD.

Previous studies revealed that anhedonia was associated with overactivation of the inflammatory response system and altered levels of inflammatory cytokines. Early animal studies found that acute systemic administration of proinflammatory factors could induce anhedonic-like behaviors in mice (15). A recent study in a rat model of spared nerve injury demonstrated that inflammatory cytokines, including interleukin-1 beta (IL-1β), interleukin-6 (IL-6) and tumor necrosis factor alpha (TNF-α), would promote anhedonia susceptibility (16). Meanwhile, the effects of interleukin-6 neutralizing antibodies have manifested symptoms of depressed mood and anhedonia in patients with rheumatoid arthritis and multicentric Castleman's disease (17). Anhedonia was reported to be associated with inflammatory gene expression in MDD as well as in cocaine use disorder (18, 19). Tang et al. (20) investigated the association between inflammatory characteristics and anhedonia in drug-naïve major depressive disorder and found that MDD patients with anhedonia showed increased levels of IL-6 and complement Factor H compared to patients without anhedonia. This evidence suggests the important role of inflammatory factors in anhedonia.

Meanwhile, anhedonia has been demonstrated to be associated with impairments of the brain reward circuits, including structural abnormalities in reward-related brain regions such as the ventral striatum, nucleus accumbens (NAcc), prefrontal cortex, amygdala, and posterior cingulate. In fact, abnormalities in these brain structures have been shown to reduce the sensitivity of subjective pleasure (21). A recent study with a large population-based sample of 19,592 people reported that reduced total gray matter volume (GMV) in multiple brain structures, including reward-related circuits, were associated with the phenotype of anhedonia and its genetic risk (22). Furthermore, anhedonia has also been found to be associated with reduced caudate (23) and NAcc volumes (24, 25) in nonclinical samples. In MDD, the severity of anhedonic symptoms was associated with reduced bilateral caudate volume (26).

Taken together, these findings suggest that both aberrant inflammation and altered brain structure may be involved in the pathophysiology of anhedonia. Previous animal and clinical studies revealed that brain structure would change after acute and chronic stimulated peripheral infection, and these changes were associated with proinflammatory cytokine levels (27, 28). In addition, increased proinflammatory cytokines were reported to be associated with brain GMV reductions in neuropsychiatric disorders, such as schizophrenia (29), MDD (30), and bipolar disorder (31). Evidence from postmortem human brains of patients with schizophrenia indicated that overexpression of IL-6 and IL-1β mRNA levels were significantly associated with brain volume reduction (32). Tsai et al. (33) reported that persistent inflammation was associated with reduction of hippocampal and GMVs in older patients with bipolar disorder. As mentioned above, although MDD patients with anhedonia were more likely to present increased inflammatory activity, the relationship between inflammation and brain structural alterations in MDD patients with anhedonia was less revealed. In this context, we examined the concentrations of plasma IL-1β, IL-6 and TNF-α, analyzed GMVs using whole-brain voxel-based analyses in outpatients with MDD, and finally explored the interrelationships between these biological determinants in MDD patients with anhedonia. Based on previous evidence, we hypothesized that there could be a link between elevated inflammatory factors and brain structural atrophy in MDD patients with anhedonia.

## Methods

### Participants

A total of forty-two MDD patients from Department of Psychiatry, The First Affiliated Hospital, Zhejiang University School of Medicine were enrolled. All subjects satisfied the Diagnostic and Statistical Manual of Mental Disorders, IV Edition (DSM-IV) criteria for MDD, as screened with Structured Clinical Interview for DSM-IV (SCID) and met the following criteria: (1) aged 18-45 years; (2) drug-naïve patients with first-episode depression or recurrent depression with continued withdrawal of >3 months; and (3) a total score of 17-item Hamilton Depression Scale (HAMD-17) ≥ 17. In this study, patients with MDD were divided into two groups, MDD with anhedonia and MDD without anhedonia. For assignment to the MDD with anhedonia group, MDD patients must have been experiencing anhedonia according to the Item 2 (loss of interest or pleasure) of the symptom criteria (A) for MDD in the DSM-IV and the threshold of transformed score of Snaith-Hamilton Pleasure Scale (SHAPS). Meanwhile, a total of 20 sex- and age-matched healthy controls (HCs) were recruited from local residents, hospital staffs and students. All HCs were thoroughly interviewed and were free from any current or lifetime history of psychiatric disorders according to the DSM IV criteria. The general exclusion criteria for all subjects were as follows:(1) existence of any major medical disease including cardiovascular, respiratory, endocrine and neurological diseases (e.g., epilepsy, brain trauma and stroke); (2) current use of any medication that might affect the central nervous system, (3) drug or alcohol dependence or abuse; (4) female with pregnancy; (5) with histories of psychotherapy and physical therapy, (6) contraindications to MRI scan, including retractors or braces, metallic implants, and claustrophobia.

This study was approved by the local Medical Ethics Committee of The First Affiliated Hospital, Zhejiang University School of Medicine. Prior to commencement of the study, all participants provided written informed consent.

### Clinical assessment

Clinical assessments were carried out by two experienced and well-trained psychiatrists from Department of Psychiatry, The First Affiliated Hospital, Zhejiang University School of Medicine. The demographic and clinical data was collected by using a self-designed questionnaire from all the participants. Depression severity was evaluated using the 17-item Hamilton Depression Scale (HAMD-17), which is the most common clinician-rated scale for assessing the severity of depression. The symptoms of anhedonia were assessed by the Snaith–Hamilton Pleasure Scale (SHAPS), which is a self-report questionnaire (34). The Chinese version has been validated (35). A total of 14 items are included in this scale, and the scale covers four categories of pleasurable activities, namely, interests and pastimes, sensory experiences, social interactions, and diet. The responses to each item are rated as either a 0 or a 1, or 0 for “Agree” or “Strongly Agree” and 1 for “Disagree” or “Strongly Disagree”. The total SHAPS score indicates the severity of anhedonia status, and a sum of transformed scores > 5 indicates the presence of severe anhedonia, which is a method that has been used as the anhedonic or nonanhedonic grouping criteria in previous studies (12, 36).

### Plasma inflammatory factor measurement

Fasting venous blood was collected from each subject in the morning, and whole blood samples were centrifuged at 3,000 rpm for 20 min. Plasma was separated and stored at −80 °C for simultaneous analysis. The concentrations of IL-1β, IL-6 and TNF-α in plasma were measured by Duoset human ELISA Kits (IL-1β: HSLB00D, R&D Systems, Minneapolis, MN, USA; IL-6: HS60DC, R&D Systems, Minneapolis, MN, USA; TNF-α: HSTA00E, R&D Systems, Minneapolis, MN, USA) according to the manufacturers' instructions. The final results are presented in pg/ml.

### MRI acquisition

Imaging data were acquired on a 3.0-T scanner (Signa, HDxt, GE healthcare, USA) with a standard birdcage head coil in the Magnetic Resonance Center at The First Affiliated Hospital, Zhejiang University School of Medicine. All participants were instructed to lie still with their eyes closed and to avoid falling asleep. The protocol in the present study involved 3D T1-weighted structural images, which were acquired by a brain volume (BRAVO) sequence with the following parameters: TR = minimum (7.3 ms), TE = minimum (3.0 ms), TI = 1,100 ms, flip angle = 7, FOV = 256 ^*^ 256 mm^2^, matrix = 256 ^*^ 256, slice thickness = 1 mm, bandwidth = 31.25 KHz, NEX = 1, slices = 192.

### Voxel-based morphometry (VBM) analysis

To analyze the structural data, we used the voxel-based morphometry (VBM) approach provided in the SPM12 software (http://fil.ion.ucl.ac.uk/spm/) of CAT12 software (http://dbm.neuro.uni-jena.de/vbm/) in the MATLAB environment. For each individual, a 3D T1-weighted image was corrected for bias-field differences and segmented into gray matter, white matter, and CSF (37) before applying DARTEL spatial normalization (38). In addition, segmentation was extended to account for partial volume effects (39) through the use of adaptive maximum a posteriori estimation (40). All scans passed standard quality checks according to an automated protocol after preprocessing, and we assessed sample homogeneity using a box plot and correlation matrix to identify outliers. Artifacts or preprocessing errors were inspected, and poor-quality scans were excluded. In the end, the images were smoothed with an 8-mm full-width Gaussian kernel at half-maximum (FWHM), and an absolute gray-matter threshold of 0.1 was applied to the entire gray/white matter margin to remove artifacts created by, for example, incorrect voxel classification.

### Statistical analysis

The analyses of demographic, clinical, and biochemical data were performed with the Statistical Package for the Social Sciences (SPSS) (version 16.0, SPSS Inc., Chicago, IL, USA). Categorical variables are expressed as percentages (m/n), and the differences among the three groups were analyzed using Chi-square tests (χ^2^). The continuous variables included in this study appeared to be distributed normally based on the normal distribution test and are represented as the mean (standard deviation), and the differences among the three groups were analyzed by one-way analysis of variance (ANOVA). Benjamini-Hochberg method (BH) was used for false discovery rate (FDR) correction to adjust the *p*-values in multi-comparison. Moreover, the effect size of ANOVA was expressed as $\eta_{p}^{2}$. Pearson correlation analysis was used to analyze the associations between the clinical data and those of biochemical factors. In our analysis, a two-sided *p* < 0.05 significance level was considered significant.

GMVs on the individual imaging maps were analyzed with one-way ANOVA and *post hoc* analyses in a voxel-by-voxel manner for the intergroup differences. In addition, potential confounders, such as, age, sex, education years, and total internal volume (TIV) were included as covariates. The significance level was set at *p* < 0.05 and corrected according to Gaussian random field (GRF) theory (voxel significance: *p* < 0.001, cluster significance: *p* < 0.05) for multiple comparisons with REST software. Finally, to evaluate any correlations between inflammation data and gray matter structural changes in MDD patients with anhedonia, whole brain multiple regression analyses integrated in SPM basic models were performed at *p* < 0.05 (GRF corrected).

## Results

### Demographic and clinical characteristics

The demographic and clinical features of all subjects are summarized in Table 1. No significant differences were observed with respect to age, sex, and education years among three groups (*p* > 0.05). The two groups of MDD patients did not differ on means of illness duration and HAMD-17 scores (*p* > 0.05). As we would expect, MDD patients with anhedonia exhibited increased SHAPS scores relative to those without anhedonia (*p* < 0.05).

**Table 1:** Demographic and clinical characteristics for all subjects (*n* = 62).

	**MDD with anhedonia, *n* = 22 means (SD)**	**MDD without anhedonia, *n* = 20 means (SD)**	**HCs, *n* = 20 means (SD)**	**Analysis *F/χ^2^***	***p*-values**
Age (years)	28.6 (7.73)	29.1 (7.70)	27.7 (5.07)	0.223	0.801
Gender (Male/Female)	5/17	10/10	10/10	4.387	0.112
Education years	14.4 (2.02)	14.4 (3.05)	15.3 (1.21)	0.985	0.379
Illness duration (months)	23.4 (27.5)	19.9 (22.9)		0.194	0.662
SHAPS score	37.7 (7.09)	26.7 (3.76)		38.14	0.000
HAMD score	25.2 (3.89)	23.7 (2.79)		2.097	0.155

HAMD, Hamilton Depression Scale; HCs, Healthy Controls; MDD, Major Depressive Disorder; SD, Standard Deviation; SHAPS, Snaith-Hamilton Pleasure Scale.

### Plasma levels of inflammatory factors among the three groups

Table 2 presents the plasma levels of inflammatory factors for three groups. Significant differences on plasma levels of IL-1β and IL-6 among three groups were identified (*p* < 0.05). Compared with HCs, both groups of MDD patients showed higher plasma levels of IL-1β (*p* < 0.05), regardless of anhedonia, however, there was no significant difference between two groups of MDD patients (*p* > 0.05). MDD patients with anhedonia showed higher plasma levels of IL-6 than HCs (*p* < 0.05), while no significant difference was found between MDD patients without anhedonia and HCs, either the two groups of MDD patients. No significant differences were found on plasma levels of TNF-α among three groups (*p* > 0.05).

**Table 2:** Comparisons of plasma levels of inflammatory factors among three groups (*n* = 62).

	**MDD with anhedonia, *n* = 22 means (SD)**	**MDD without anhedonia, *n* = 20 means (SD)**	**HCs, *n* = 20 means (SD)**	**Analysis *F***	***p*-values**	**Adjusted *p-*values**	** $\eta_{p}^{2}$ **
IL-1β (pg/ml)	767.0 (345.7)*^*a*^*	708.4 (473.1)*^*a*^*	402.5 (186.7)	6.240	0.003	0.009	0.175
IL-6 (pg/ml)	948.4 (387.4)*^*a*^*	708.2 (379.4)	594.7 (482.8)	3.947	0.025	0.037	0.118
TNF-α (pg/ml)	499.1 (225.2)	437.2 (221.4)	328.7 (260.6)	2.776	0.070	0.070	0.086

MDD, Major Depressive Disorder; HCs, Healthy Controls; SD, Standard Deviation; IL, Interleukin; TNF, Tumor Necrosis Factor.

^a^Compared with HCs, p < 0.01.

### Alterations in GMVs

**Figure 1:**
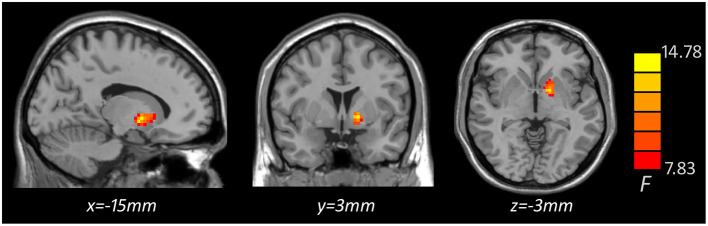
Brain region showing significant structural difference among three groups (*p* < 0.05, *GRF corrected*). The color scale represents *F* values of ANOVA.

**Figure 2:**
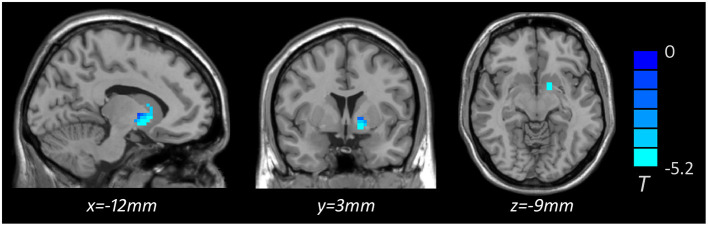
Brain region showing significant structural difference between MDD with anhedonia and HCs (*p* < 0.05, *GRF corrected*). The color scale represents *t* values.

**Figure 3:**
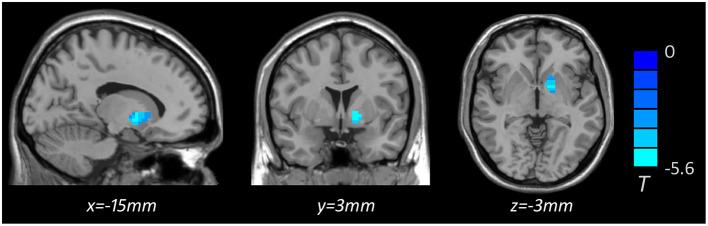
Brain region showing significant structural difference between MDD with and without anhedonia (*p* < 0.05, *GRF corrected*). The color scale represents *t* values.

Based on one-way ANOVA, significant GMV difference in the left lateral globus pallidus (LGP) was observed among three groups (Table 3, Figure 1). *Post hoc* pairwise comparisons revealed that MDD patients with anhedonia exhibited decreased GMV in the left putamen when comparing to HCs (Table 4, Figure 2). Meanwhile, MDD with anhedonia also showed reduced GMV in the left LGP when compared with MDD without anhedonia (Table 4, Figure 3). However, no significant difference was found between MDD patients without anhedonia and HCs.

**Table 3:** Brain region showing significant structural difference among three groups (*p* < 0.05, *GRF corrected*).

**Brain region**	**Hemisphere**	**Cluster size**	***F* value**	**MNI coordinate**		
				***x***	***y***	***z***
LGP	L	63	14.73	−15	3	−3

LGP, Lateral Globus Pallidus; MNI, Montreal Neurological Institute.

**Table 4:** Brain region showing inter-group differences based on the results of ANOVA (*p* < 0.05, *GRF corrected*).

**Brain region**	**Hemisphere**	**Cluster size**	***t* value**	**MNI coordinate**		
				***x***	***y***	***z***
**MDD with anhedonia**<**HCs**						
Putamen	L	48	−5.150	−12	3	−9
**MDD with**<**without anhedonia**						
LGP	L	50	−5.582	−15	3	−3

HCs, Healthy Controls; LGP, Lateral Globus Pallidus; MDD, Major Depressive Disorder; MNI, Montreal Neurological Institute.

### Correlation analyses

**Figure 4:**
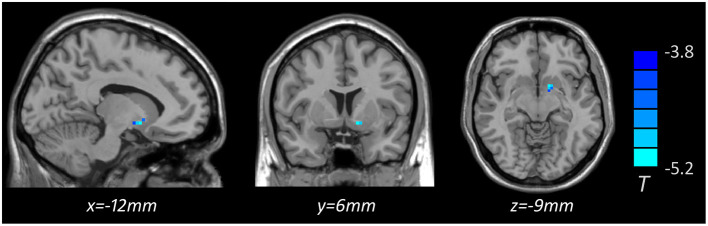
Brain region showing decreased gray matter volume associated with plasma level of IL-6 (pg/ml) in MDD with anhedonia (*p* < 0.05, *GRF corrected*). The color scale represents *t* values.

Voxel-based whole-brain correlation analysis showed that GMV in the left putamen was negatively associated with plasma levels of IL-6 in MDD patients with anhedonia (Table 5, Figure 4), while this association was not significant in other two groups. In addition, a positive correlation was observed between plasma levels of IL-6 and total scores of SHAPS (*r* = 0.421, *p* = 0.005) in patients with MDD. Nevertheless, there was no significant association of plasma levels of IL-1β or TNF-α with total scores of SHAPS in MDD patients.

**Table 5:** Region of decreased gray matter volume in MDD with anhedonia correlated to plasma level of IL-6 (pg/ml), *p* < 0.05, *GRF corrected*.

**Brain region**	**Hemisphere**	**Cluster size**	***t* value**	**MNI coordinate**		
				***x***	***y***	***z***
Putamen	L	15	−5.159	−12	6	−9

MDD, Major Depressive Disorder; MNI, Montreal Neurological Institute.

## Discussion

The present study investigated the difference in inflammatory factor levels and GMV between MDD patients with and without anhedonia and explored the association between inflammatory factor concentrations and morphometric alterations in MDD patients with anhedonia. To our knowledge, this is the first study to investigate interrelationships between GMV changes and plasma levels of inflammatory factors in MDD patients with anhedonic symptoms. Our results demonstrated that plasma levels of IL-6 in MDD patients with anhedonia, but not in MDD patients without anhedonia, were higher than those in HCs, which were also positively correlated with SHAPS scores in MDD patients. More importantly, VBM analyses revealed that MDD patients with anhedonia exhibited reduced GMV in the left putamen and LGP and showed a significantly negative association between IL-6 levels and the left LGP GMV. Our results emphasize the importance of focusing on the influence of inflammatory cytokine concentrations and morphometric alterations in MDD patients with anhedonia, which may provide a new perspective for the pathophysiology of anhedonia and depression.

In the present study, significant differences on plasma levels of IL-1β and IL-6 were found among the two groups of MDD patients and HCs, specifically, patients with MDD showed higher plasma levels of IL-1β and IL-6 than HCs, which was consistent with many studies assessing the association between depression and inflammatory markers. More interestingly, we found that the plasma levels of IL-6 were significantly elevated in MDD patients with anhedonia (but not in MDD patients without anhedonia) compared to HCs, and the correlation analysis showed a significantly positive correlation between the IL-6 levels and the severity of anhedonia. Despite the relatively few clinical reports in this area of interest, some previous studies were consistent with and supported our findings. A large-scale cohort study from the UK Biobank and Netherlands Study of Depression and Anxiety revealed an association between IL-6 and anhedonia (41). Similarly, IL-6 was proven to be positively associated with SHAPS scores with an exploratory sensitivity analysis in depressed adolescents (42). Tang et al. (20) also reported that patients with anhedonia showed higher levels of IL-6 than those without anhedonia in drug-naïve major depressive disorder, although they failed to find altered levels of IL-6 between depressive patients and healthy subjects. Meanwhile, some animal studies demonstrated that IL-6 promotes anhedonia susceptibility in spared nerve injury (16) and that IL-6 receptor antagonists attenuate postpartum anhedonia in female rats (43). IL-6 levels play a crucial role in the pathogenesis of depression (44) and have been linked to depression prognosis (45, 46) and treatment outcomes (47, 48). Some scholars supported the idea that the symptoms and subtypes of depression influence IL-6 levels and suggested that using other depression-related cytokines or their receptors as biomarkers may help to classify MDD biological subtypes (49, 50). Rudolf et al. (51) found that peripheral IL-6 levels were elevated in atypical MDD patients but not in typical MDD patients when compared with controls. Hence, we speculated that the altered IL-6 levels may be associated with anhedonic features in MDD. Thus, more investigation is needed in the future.

With respect to neuroimaging findings in the present study, the main influence of anhedonia was discovered in the striatum, with reduced GMV in the left putamen and LGP. The striatum, which is composed of the putamen, globus pallidus and caudate nucleus, is the primary input structure of the basal ganglia and plays a key role in reward-related activities. Similar to emphasizing the prominent role of anhedonia in MDD clinical symptoms, robust gray matter reduction in the striatum was reported in MDD (52). A meta-analysis of a magnetic resonance imaging study revealed that patients with MDD showed reduced GMVs in the putamen relative to healthy individuals (53). Auerbach et al. (24) found that the smaller volume in striatal regions prospectively predicted anhedonia severity in adolescents with MDD. Meanwhile, the hypofunction in the ventral striatal and orbitofrontal regions during unexpected reward receipt was proven to be associated with anhedonia in MDD and schizophrenia, which suggested that abnormal frontostriatal activity may underlie anhedonic symptoms in depression and schizophrenia (54). In addition, some associations between striatum and suicidal risk and poor treatment results in MDD patients were found. A study reported that reduced bilateral putamen and left caudate GMV significantly predicted scores of the death version of the Implicit Association Test (IAT) in adolescents, which suggested that higher suicide risk was associated with limited GMV in the striatum (55). Downar et al. reported that MDD patients with no response to dorsomedial prefrontal repetitive transcranial magnetic stimulation showed aberrant connectivity of the striatum (56), and the effect of electroconvulsive therapy on striatal morphometry was discovered in MDD (57). As anhedonia is regarded as a strong predictor for increased suicidal behavior and poor response to antidepressant treatment, these overlapping findings support the possible role of reduced striatal volume in the pathogenesis of anhedonia in MDD.

Moreover, the present study showed a significant negative relationship between plasma IL-6 levels and GMV in the LGP in MDD patients with anhedonia using whole-brain voxel-based correlation analysis. Previous works demonstrated that acute systemic inflammation could induce decreased striatal reactivity and disturb neural processes involving motivation and loss of interest (58). The potential mechanisms involve the effect of inflammation on dopamine synthesis, release, and reuptake, increased synaptic and additional synaptic glutamate, and activation of kynurenine pathway metabolites^.^, which may influence the reward related neural circuitry and induce the symptom of anhedonia (59–61). Eisenberger et al. (58) reported that low-dose endotoxin was associated with increased depressive mood and reduced ventral striatal response to reward cues in healthy participants. Burrows et al. (62) found that the lower activation of striatal activity was negatively correlated with serum CRP levels in MDD patients in the monetary incentive delay task. Meanwhile, the serum ratio of kynurenine/tryptophan, which presents an enzyme activated by proinflammatory cytokines and is involved in the synthesis of kynurenine from tryptophan, was reported to be negatively correlated with striatal volume (63). These previous findings supported our results and confirmed the complex relationship between the inflammatory state and structural alterations of the brain, and we suspected that IL-6 may be a key biological marker associated with the striatal volume for anhedonia in MDD.

## Limitation

The present study had certain limitations. First, the sample size in this study was relatively small, which restricted the statistical power and generalization of the findings. Second, this study was a cross-sectional design and was unable to explore dynamic changes in inflammatory factors and brain structure, precluding causal inferences. Third, although the distinctions of different aspects of anhedonia on brain structure and inflammation have been revealed (64, 65), the present study did not investigate the effects of different aspects of anhedonia due to the modest sample size. Finally, only three inflammatory cytokines, including IL-1β, IL-6, and TNF-α, were measured in this study, consequently, the findings of inflammatory characteristics in MDD patients with anhedonia were quite limited. In this context, future longitudinal studies containing the abovementioned aspects may be helpful to elucidate the detailed pathway underlying inflammation-related brain alterations in MDD patients with anhedonia. Additionally, animal studies should further explore whether inflammatory factors are causally related to neural changes and subsequently anhedonia.

## Conclusion

In summary, the present research outcomes suggest that anhedonia is associated with increased plasma levels of IL-6 and decreased GMV in the left striatal structures. In addition, this study demonstrates that GMV loss in the left putamen is related to increased plasma levels of IL-6 in MDD with anhedonia, which provides further insights into the possible mechanisms of anhedonia.

## Data availability statement

The original contributions presented in the study are included in the article/supplementary material, further inquiries can be directed to the corresponding author/s.

## Ethics statement

The studies involving human participants were reviewed and approved by the Local Medical Ethics Committee of The First Affiliated Hospital, Zhejiang University School of Medicine. The patients/participants provided their written informed consent to participate in this study.

## Author contributions

SL and CW designed the study, conducted the statistical analyses, and wrote the first draft of the manuscript. LJ and ZF finished the clinical assessments. SH recruited the sample. JL and TM tested and stored the blood samples. HH, MH, and YX designed the study and had full access to all of the data in the study and took responsibility for the integrity of the data and the accuracy of the data analysis. All authors contributed to and have approved the final manuscript.

## Funding

This work was supported by the National Natural Science Foundation of China (82071521 to SL) and the Natural Science Foundation of Zhejiang Province (LY19H090017 to SL).

## Conflict of interest

The authors declare that the research was conducted in the absence of any commercial or financial relationships that could be construed as a potential conflict of interest.

## Publisher's note

All claims expressed in this article are solely those of the authors and do not necessarily represent those of their affiliated organizations, or those of the publisher, the editors and the reviewers. Any product that may be evaluated in this article, or claim that may be made by its manufacturer, is not guaranteed or endorsed by the publisher.

## References

[1] Zhdanava M Kuvadia H Joshi K Daly E Pilon D Rossi C. Economic burden of treatment-resistant depression in privately insured us patients with physical conditions. J Manag Care Spec Pharm. (2020) 26:996–1007. 10.18553/jmcp.2020.20017 32552362 PMC10391320

[2] Huang Y Wang Y Wang H Liu Z Yu X Yan J. Prevalence of mental disorders in china: a cross-sectional epidemiological study. Lancet Psychiat. (2019) 6:211–24. 10.1016/S2215-0366(18)30511-X 30792114

[3] Soleimani L Lapidus KA Iosifescu DV. Diagnosis and treatment of major depressive disorder. Neurologic Clini. (2011) 29:177–93. 10.1016/j.ncl.2010.10.010 21172578

[4] Kennis M Gerritsen L van Dalen M Williams A Cuijpers P Bockting C. Prospective biomarkers of major depressive disorder: a systematic review and meta-analysis. Mol Psychiatry. (2020) 25:321–38. 10.1038/s41380-019-0585-z 31745238 PMC6974432

[5] Troubat R Barone P Leman S Desmidt T Cressant A Atanasova B. Neuroinflammation and depression: a review. Eur J Neurosci. (2021) 53:151–71. 10.1111/ejn.14720 32150310

[6] Gong Q He Y. Depression, neuroimaging and connectomics: a selective overview. Biol Psychiatry. (2015) 77:223–35. 10.1016/j.biopsych.2014.08.009 25444171

[7] Zhang FF Peng W Sweeney JA Jia ZY Gong QY. Brain structure alterations in depression: psychoradiological evidence. CNS Neurosci Ther. (2018) 24:994–1003. 10.1111/cns.12835 29508560 PMC6489983

[8] Uher R Perlis RH Henigsberg N Zobel A Rietschel M Mors O. Depression symptom dimensions as predictors of antidepressant treatment outcome: replicable evidence for interest-activity symptoms. Psychol Med. (2012) 42:967–80. 10.1017/S0033291711001905 21929846 PMC3787526

[9] Ducasse D Loas G Dassa D Gramaglia C Zeppegno P Guillaume S. Anhedonia is associated with suicidal ideation independently of depression: a meta-analysis. Depress Anxiety. (2018) 35:382–92. 10.1002/da.22709 29232491

[10] Buckner JD Joiner TE. Jr. Pettit JW Lewinsohn PM Schmidt NB. Implications of the Dsm's emphasis on sadness and anhedonia in major depressive disorder. Psychiatry Res. (2008) 159:25–30. 10.1016/j.psychres.2007.05.010 18334272 PMC3688280

[11] Cooper JA Arulpragasam AR Treadway MT. Anhedonia in depression: biological mechanisms and computational models. Curr Opin Behav Sci. (2018) 22:128–35. 10.1016/j.cobeha.2018.01.024 29503842 PMC5828520

[12] Vinckier F Gourion D Mouchabac S. Anhedonia predicts poor psychosocial functioning: results from a large cohort of patients treated for major depressive disorder by general practitioners. Eur Psychiatry. (2017) 44:1–8. 10.1016/j.eurpsy.2017.02.485 28535406

[13] Treadway MT Zald DH. Reconsidering anhedonia in depression: lessons from translational neuroscience. Neurosci Biobehav Rev. (2011) 35:537–55. 10.1016/j.neubiorev.2010.06.006 20603146 PMC3005986

[14] Hasler G Drevets WC Manji HK Charney DS. Discovering endophenotypes for major depression. Neuropsychopharmacology. (2004) 29:1765–81. 10.1038/sj.npp.1300506 15213704

[15] Salazar A Gonzalez-Rivera BL Redus L Parrott JM O'Connor JC. Indoleamine 2,3-dioxygenase mediates anhedonia and anxiety-like behaviors caused by peripheral lipopolysaccharide immune challenge. Horm Behav. (2012) 62:202–9. 10.1016/j.yhbeh.2012.03.010 22504306 PMC3425718

[16] Fang X Zhan G Zhang J Xu H Zhu B Hu Y. Abnormalities in inflammatory cytokines confer susceptible to chronic neuropathic pain-related anhedonia in a rat model of spared nerve injury. Clin Psychopharmacol Neurosci. (2019) 17:189–99. 10.9758/cpn.2019.17.2.189 30905119 PMC6478091

[17] Sun Y Wang D Salvadore G Hsu B Curran M Casper C. The effects of interleukin-6 neutralizing antibodies on symptoms of depressed mood and anhedonia in patients with rheumatoid arthritis and multicentric castleman's disease. Brain Behav Immun. (2017) 66:156–64. 10.1016/j.bbi.2017.06.014 28676350

[18] Bekhbat M Treadway MT Goldsmith DR Woolwine BJ Haroon E Miller AH. Gene signatures in peripheral blood immune cells related to insulin resistance and low tyrosine metabolism define a sub-type of depression with high crp and anhedonia. Brain Behav Immun. (2020) 88:161–5. 10.1016/j.bbi.2020.03.015 32198016 PMC7415632

[19] Fries GR Khan S Stamatovich S Dyukova E Walss-Bass C Lane SD. Anhedonia in cocaine use disorder is associated with inflammatory gene expression. PLoS ONE. (2018) 13:e0207231. 10.1371/journal.pone.0207231 30408130 PMC6224118

[20] Tang W Liu H Chen L Zhao K Zhang Y Zheng K. Inflammatory cytokines, complement factor H and anhedonia in drug-naive major depressive disorder. Brain Behav Immun. (2021) 95:238–44. 10.1016/j.bbi.2021.03.022 33794316

[21] Martins D Rademacher L Gabay AS Taylor R Richey JA Smith DV. Mapping social reward and punishment processing in the human brain: a voxel-based meta-analysis of neuroimaging findings using the social incentive delay task. Neurosci Biobehav Rev. (2021) 122:1–17. 10.1016/j.neubiorev.2020.12.034 33421544

[22] Zhu X Ward J Cullen B Lyall DM Strawbridge RJ Lyall LM. Phenotypic and genetic associations between anhedonia and brain structure in UK biobank. Transl Psychiatry. (2021) 11:395. 10.1038/s41398-021-01522-4 34282121 PMC8289859

[23] Harvey PO Pruessner J Czechowska Y Lepage M. Individual differences in trait anhedonia: a structural and functional magnetic resonance imaging study in non-clinical subjects. Mol Psychiatry. (2007) 12:67–75. 10.1038/sj.mp.4002021 17505465

[24] Auerbach RP Pisoni A Bondy E Kumar P Stewart JG Yendiki A. Neuroanatomical prediction of anhedonia in adolescents. Neuropsychopharmacology. (2017) 42:2087–95. 10.1038/npp.2017.28 28165037 PMC5561341

[25] Wacker J Dillon DG Pizzagalli DA. The role of the nucleus accumbens and rostral anterior cingulate cortex in anhedonia: integration of resting EEG, FMRI, and volumetric techniques. Neuroimage. (2009) 46:327–37. 10.1016/j.neuroimage.2009.01.058 19457367 PMC2686061

[26] Pizzagalli DA Holmes AJ Dillon DG Goetz EL Birk JL Bogdan R. Reduced caudate and nucleus accumbens response to rewards in unmedicated individuals with major depressive disorder. Am J Psychiatry. (2009) 166:702–10. 10.1176/appi.ajp.2008.08081201 19411368 PMC2735451

[27] Chen J Yan Y Yuan F Cao J Li S Eickhoff SB. Brain grey matter volume reduction and anxiety-like behavior in lipopolysaccharide-induced chronic pulmonary inflammation rats: a structural mri study with histological validation. Brain Behav Immun. (2019) 76:182–97. 10.1016/j.bbi.2018.11.020 30472482

[28] Zheng H Ford BN Bergamino M Kuplicki R Tulsa I Hunt PW. A hidden menace? Cytomegalovirus infection is associated with reduced cortical gray matter volume in major depressive disorder. Mol Psychiatry. (2021) 26:4234–44. 10.1038/s41380-020-00932-y 33223520 PMC8140068

[29] Quide Y Bortolasci CC Spolding B Kidnapillai S Watkeys OJ Cohen-Woods S. Systemic inflammation and grey matter volume in schizophrenia and bipolar disorder: moderation by childhood trauma severity. Prog Neuropsychopharmacol Biol Psychiatry. (2021) 105:110013. 10.1016/j.pnpbp.2020.110013 32540496

[30] Han KM Ham BJ. How inflammation affects the brain in depression: a review of functional and structural MRI studies. J Clin Neurol. (2021) 17:503–15. 10.3988/jcn.2021.17.4.503 34595858 PMC8490908

[31] Chen MH Kao ZK Chang WC Tu PC Hsu JW Huang KL. Increased proinflammatory cytokines, executive dysfunction, and reduced gray matter volumes in first-episode bipolar disorder and major depressive disorder. J Affect Disord. (2020) 274:825–31. 10.1016/j.jad.2020.05.158 32664021

[32] Zhang Y Catts VS Sheedy D McCrossin T Kril JJ Shannon Weickert C. Cortical grey matter volume reduction in people with schizophrenia is associated with neuro-inflammation. Transl Psychiatry. (2016) 6:e982. 10.1038/tp.2016.238 27959331 PMC5290336

[33] Tsai SY Gildengers AG Hsu JL Chung KH Chen PH Huang YJ. Inflammation associated with volume reduction in the gray matter and hippocampus of older patients with bipolar disorder. J Affect Disord. (2019) 244:60–6. 10.1016/j.jad.2018.10.093 30317016

[34] Nakonezny PA Morris DW Greer TL Byerly MJ Carmody TJ Grannemann BD. Evaluation of anhedonia with the snaith-hamilton pleasure scale (Shaps) in adult outpatients with major depressive disorder. J Psychiatr Res. (2015) 65:124–30. 10.1016/j.jpsychires.2015.03.010 25864641 PMC7505238

[35] Liu WH Wang LZ Zhu YH Li MH Chan RC. Clinical utility of the snaith-hamilton-pleasure scale in the chinese settings. BMC Psychiatry. (2012) 12:184. 10.1186/1471-244X-12-184 23110667 PMC3549923

[36] Wu C Lu J Lu S Huang M Xu Y. Increased ratio of mature BDNF to precursor-bdnf in patients with major depressive disorder with severe anhedonia. J Psychiatr Res. (2020) 126:92–7. 10.1016/j.jpsychires.2020.05.010 32428748

[37] Ashburner J Friston KJ. Unified segmentation. Neuroimage. (2005) 26:839–51. 10.1016/j.neuroimage.2005.02.018 15955494

[38] Ashburner J A. Fast Diffeomorphic image registration algorithm. Neuroimage. (2007) 38:95–113. 10.1016/j.neuroimage.2007.07.007 17761438

[39] Tohka J Zijdenbos A Evans A. Fast and robust parameter estimation for statistical partial volume models in brain MRI. Neuroimage. (2004) 23:84–97. 10.1016/j.neuroimage.2004.05.007 15325355

[40] Jukes TH. How many anticodons? Science. (1977) 198:319–20. 10.1126/science.910132 910132

[41] Milaneschi Y Kappelmann N Ye Z Lamers F Moser S Jones PB. Association of inflammation with depression and anxiety: evidence for symptom-specificity and potential causality from UK biobank and nesda cohorts. Mol Psychiatry. (2021) 26:7393–402. 10.1038/s41380-021-01188-w 34135474 PMC8873022

[42] Rengasamy M Marsland A McClain L Kovats T Walko T Pan L. Longitudinal relationships of cytokines, depression and anhedonia in depressed adolescents. Brain Behav Immun. (2021) 91:74–80. 10.1016/j.bbi.2020.09.004 32919038 PMC7952030

[43] Gomez J Haas NA Schwarz JM. An Il-6 receptor antagonist attenuates postpartum anhedonia, but has no effect on anhedonia precipitated by subchronic stress in female rats. Psychopharmacology (Berl). (2019) 236:2983–95. 10.1007/s00213-019-05194-3 30830260

[44] Zhang JC Yao W Dong C Yang C Ren Q Ma M. Blockade of interleukin-6 receptor in the periphery promotes rapid and sustained antidepressant actions: a possible role of gut-microbiota-brain axis. Transl Psychiatry. (2017) 7:e1138. 10.1038/tp.2017.112 28556833 PMC5534942

[45] Kelly KM Smith JA Mezuk B. Depression and interleukin-6 signaling: a mendelian randomization study. Brain Behav Immun. (2021) 95:106–14. 10.1016/j.bbi.2021.02.019 33631287 PMC11081733

[46] Roohi E Jaafari N Hashemian F. On inflammatory hypothesis of depression: what is the role of IL-6 in the middle of the chaos? J Neuroinflammation. (2021) 18:45. 10.1186/s12974-021-02100-7 33593388 PMC7884972

[47] Kang HJ Bae KY Kim SW Kim JT Park MS Cho KH. Effects of interleukin-6, interleukin-18, and statin use, evaluated at acute stroke, on post-stroke depression during 1-year follow-up. Psychoneuroendocrinology. (2016) 72:156–60. 10.1016/j.psyneuen.2016.07.001 27428088

[48] Carboni L McCarthy DJ Delafont B Filosi M Ivanchenko E Ratti E. Biomarkers for response in major depression: comparing paroxetine and venlafaxine from two randomised placebo-controlled clinical studies. Transl Psychiatry. (2019) 9:182. 10.1038/s41398-019-0521-7 31375659 PMC6677721

[49] Wang M Wei J Yang X Ni P Wang Y Zhao L. The level of Il-6 was associated with sleep disturbances in patients with major depressive disorder. Neuropsychiatr Dis Treat. (2019) 15:1695–700. 10.2147/NDT.S202329 31417262 PMC6602297

[50] Ting EY Yang AC Tsai SJ. Role of interleukin-6 in depressive disorder. Int J Mol Sci. (2020) 21. 10.3390/ijms21062194 32235786 PMC7139933

[51] Rudolf S Greggersen W Kahl KG Huppe M Schweiger U. Elevated Il-6 levels in patients with atypical depression but not in patients with typical depression. Psychiatry Res. (2014) 217:34–8. 10.1016/j.psychres.2014.02.016 24673855

[52] Zheng R Zhang Y Yang Z Han S Cheng J. Reduced brain gray matter volume in patients with first-episode major depressive disorder: a quantitative meta-analysis. Front Psychiatry. (2021) 12:671348. 10.3389/fpsyt.2021.671348 34276443 PMC8282212

[53] Koolschijn PC van Haren NE Lensvelt-Mulders GJ Hulshoff Pol HE Kahn RS. Brain volume abnormalities in major depressive disorder: a meta-analysis of magnetic resonance imaging studies. Hum Brain Mapp. (2009) 30:3719–35. 10.1002/hbm.20801 19441021 PMC6871089

[54] Segarra N Metastasio A Ziauddeen H Spencer J Reinders NR Dudas RB. Abnormal frontostriatal activity during unexpected reward receipt in depression and schizophrenia: relationship to anhedonia. Neuropsychopharmacology. (2016) 41:2001–10. 10.1038/npp.2015.370 26708106 PMC4820052

[55] Ho TC Cichocki AC Gifuni AJ Catalina Camacho M Ordaz SJ Singh MK. Reduced dorsal striatal gray matter volume predicts implicit suicidal ideation in adolescents. Soc Cogn Affect Neurosci. (2018) 13:1215–24. 10.1093/scan/nsy089 30256980 PMC6234322

[56] Downar J Geraci J Salomons TV Dunlop K Wheeler S McAndrews MP. Anhedonia and reward-circuit connectivity distinguish nonresponders from responders to dorsomedial prefrontal repetitive transcranial magnetic stimulation in major depression. Biol Psychiatry. (2014) 76:176–85. 10.1016/j.biopsych.2013.10.026 24388670

[57] Wade BS Joshi SH Njau S Leaver AM Vasavada M Woods RP. Effect of Electroconvulsive therapy on striatal morphometry in major depressive disorder. Neuropsychopharmacology. (2016) 41:2481–91. 10.1038/npp.2016.48 27067127 PMC4987846

[58] Eisenberger NI Berkman ET Inagaki TK Rameson LT Mashal NM Irwin MR. Inflammation-induced anhedonia: endotoxin reduces ventral striatum responses to reward. Biol Psychiatry. (2010) 68:748–54. 10.1016/j.biopsych.2010.06.010 20719303 PMC3025604

[59] Byrne ML Whittle S Allen NB. The role of brain structure and function in the association between inflammation and depressive symptoms: a systematic review. Psychosom Med. (2016) 78:389–400. 10.1097/PSY.0000000000000311 26910795

[60] Haroon E Welle JR Woolwine BJ Goldsmith DR Baer W Patel T. Associations among peripheral and central kynurenine pathway metabolites and inflammation in depression. Neuropsychopharmacology. (2020) 45:998–1007. 10.1038/s41386-020-0607-1 31940661 PMC7162907

[61] Lucido MJ Bekhbat M Goldsmith DR Treadway MT Haroon E Felger JC. Aiding and abetting anhedonia: impact of inflammation on the brain and pharmacological implications. Pharmacol Rev. (2021) 73:1084–117. 10.1124/pharmrev.120.000043 34285088 PMC11060479

[62] Burrows K Stewart JL Kuplicki R Figueroa-Hall L Spechler PA Zheng H. Elevated peripheral inflammation is associated with attenuated striatal reward anticipation in major depressive disorder. Brain Behav Immun. (2021) 93:214–25. 10.1016/j.bbi.2021.01.016 33508469 PMC7979507

[63] Savitz J Dantzer R Meier TB Wurfel BE Victor TA McIntosh SA. Activation of the kynurenine pathway is associated with striatal volume in major depressive disorder. Psychoneuroendocrinology. (2015) 62:54–8. 10.1016/j.psyneuen.2015.07.609 26232650 PMC4637239

[64] Liu X Li L Li M Ren Z Ma P. Characterizing the subtype of anhedonia in major depressive disorder: a symptom-specific multimodal MRI study. Psychiatry Res Neuroimaging. (2021) 308:111239. 10.1016/j.pscychresns.2020.111239 33453684

[65] Nguyen TNB Ely BA Pick D Patel M Xie H Kim-Schulze S. Clenbuterol attenuates immune reaction to lipopolysaccharide and its relationship to anhedonia in adolescents. Brain Behav Immun. (2022) 106:89–99. 10.1016/j.bbi.2022.07.163 35914697 PMC9817216

